# Newborn Screening for Pompe Disease in Illinois: Experience with 684,290 Infants

**DOI:** 10.3390/ijns6010004

**Published:** 2020-01-21

**Authors:** Barbara K. Burton, Joel Charrow, George E. Hoganson, Julie Fleischer, Dorothy K. Grange, Stephen R. Braddock, Lauren Hitchins, Rachel Hickey, Katherine M. Christensen, Daniel Groepper, Heather Shryock, Pamela Smith, Rong Shao, Khaja Basheeruddin

**Affiliations:** 1 Department of Pediatrics, Feinberg School of Medicine of Northwestern University, Chicago, IL 60611, USA; JCharrow@luriechildrens.org; 2 Department of Pediatrics, Ann and Robert H. Lurie Children’s Hospital of Chicago, Chicago, IL 60611, USA; lhitchins@luriechildrens.org (L.H.); rahickey@luriechildrens.org (R.H.); 3 Department of Pediatrics, University of Illinois College of Medicine, Chicago, IL 60612, USA; geh@uic.edu; 4 Department of Pediatrics, Southern Illinois University School of Medicine, Springfield, IL 62701, USA; jfleischer95@siumed.edu (J.F.); dgroepper@siumed.edu (D.G.); 5 Department of Pediatrics, Washington University School of Medicine and St. Louis Children’s Hospital, St. Louis, MO 63110, USA; grange_d@kids.wustl.edu; 6 Department of Pediatrics, Saint Louis University, St. Louis, MO 63104, USA; braddock@slu.edu (S.R.B.); katherine.christensen@health.slu.edu (K.M.C.); 7 Office of Health Promotion, Illinois Department of Public Health, Springfield, IL 62761, USA; Heather.Shryock@Illinois.gov (H.S.); Pamela.E.Smith@illinois.gov (P.S.); 8 Newborn Screening Laboratory, Illinois Department of Public Health, Chicago, IL 60603, USA; Rong.Shao@Illinois.gov (R.S.); Khaja.Basheeruddin@Illinois.gov (K.B.)

**Keywords:** Pompe disease, newborn screening

## Abstract

Statewide newborn screening for Pompe disease began in Illinois in 2015. As of 30 September 2019, a total of 684,290 infants had been screened and 395 infants (0.06%) were screen positive. A total of 29 cases of Pompe disease were identified (3 infantile, 26 late-onset). While many of the remainder were found to have normal alpha-glucosidase activity on the follow-up testing (234 of 395), other findings included 62 carriers, 39 infants with pseudodeficiency, and eight infants who could not be given a definitive diagnosis due to inconclusive follow-up testing.

## 1. Introduction

Pompe disease is an autosomal recessive lysosomal storage disorder resulting from the deficiency of acid alpha-glucosidase (GAA). The deficiency of enzyme activity results in the lysosomal accumulation of glycogen and multisystemic clinical manifestations, including prominent skeletal muscle weakness. Patients with the most severe form of the disorder, referred to as infantile onset Pompe disease (IOPD), also have cardiac involvement manifested as hypertrophic cardiomyopathy. In the absence of treatment, patients with IOPD rarely survive beyond two years of age. Patients with late onset Pompe disease (LOPD) may develop clinical manifestations at any age from early childhood through adult life. Progressive limb girdle muscle weakness is the hallmark of the disorder, with disproportionate involvement of the respiratory muscles often leading to respiratory insufficiency. Cardiac involvement is rare in patients with LOPD. There are patients who exhibit an intermediate phenotype with onset of muscle weakness and motor delay in the first year of life without cardiomyopathy. These patients are variably referred to as having either LOPD or atypical IOPD. Enzyme replacement therapy with alglucosidase alfa has been available for both IOPD and LOPD since 2006 and has significantly changed the natural history of the disorder. In patients with IOPD, it prolongs ventilator-free survival and often results in resolution of cardiomyopathy [1]. A subset of patients with IOPD, particularly those who are cross-reacting material (CRIM) negative, are at high risk of developing high titer antibodies to the enzyme, however, with subsequent loss of efficacy [2]. Immune modulation protocols have been developed and have shown to be effective in preventing the development of high titer antibodies in these patients [3]. In patients with LOPD, treatment with alglucosidase alfa may result in improved endurance as measured on the 6-min walk test and stabilization of motor and pulmonary function [4].

Patients with IOPD invariably exhibit elevated levels of serum creatine kinase (CK), whether diagnosed clinically or through newborn screening [5]. Patients with LOPD often, but not always, have elevated CK levels as well. In all patients with elevated levels, a decline is typically observed after initiation of enzyme replacement therapy. A second biomarker that is useful in the diagnosis and monitoring of Pompe disease is a specific urinary glucose tetrasaccharide (Glc4 or Hex4) [6]. In the newborn screening setting, levels of this tetrasaccharide have been reported to be consistently elevated in patients with IOPD but normal in those with LOPD who do not require therapy prior to three years of age [7].

Pseudodeficiency for the GAA enzyme has been well-described and is particularly common in Asian populations [8]. Patients with pseudodeficiency have low levels of GAA measured in vitro, at times as low as those observed in affected individuals but have no evidence of clinical disease or glycogen storage in tissues. Common alleles associated with pseudodeficiency have been identified. 

The rapidly progressive nature of IOPD and the availability of disease-modifying therapy was the impetus for the initiation of a pilot screening program for Pompe disease in Taiwan in 2005. Newborn screening for this disorder was shown to not only be feasible [9], but to also improve the prognosis for infantile onset Pompe disease through the earlier implementation of enzyme replacement therapy [10]. Missouri became the first state in the United States to implement newborn screening for Pompe disease in 2013 using the digital microfluidic method [11]. The data from both Taiwan and Missouri were instrumental in securing the addition of Pompe disease to the Recommended Uniform Screening Panel (RUSP) in the US in 2015. 

Illinois was the second state in the United Sates to implement statewide newborn screening for Pompe disease and the first to do so using tandem mass spectrometry. Pilot screening in selected hospitals began in November 2014 and was expanded statewide in June 2015. Results from the initial 15 months of screening were previously reported [12]. The purpose of this communication was to extend the initial report and describe the outcome of Pompe newborn screening through September 2019.

## 2. Materials and Methods

**Figure 1:**
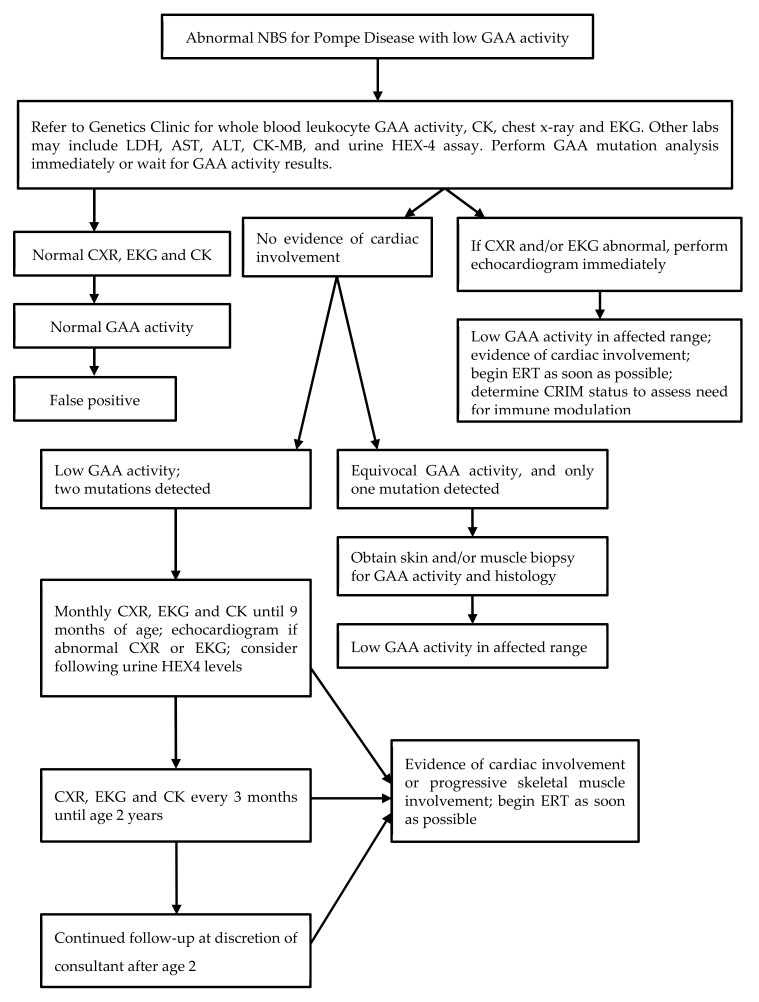
Pompe Disease Algorithm.

Newborn screening for Pompe disease is performed by determination of alpha-glucosidase enzyme activity in dried blood spots by liquid chromatography–mass spectrometry (LC–MS) using a multiplex assay with reagents from PerkinElmer^®^. The method and testing cutoffs have been previously described [12]. All infants born in the state of Illinois have been tested since June 2015. Parents can opt out of any newborn screening but cannot selectively opt out of testing for specific disorders. In practice, the opt-out option is virtually never utilized. Infants with a positive screen are urgently referred to one of several referral centers for diagnostic evaluation. A protocol for follow-up (Figure 1) was developed by a working group prior to the initiation of screening and is generally followed by all of the referral centers but is not mandatory and the specific testing ordered is at the discretion of each consulting provider. All centers obtain blood for creatinine kinase (CK) and alpha-glucosidase activity at the time of initial assessment, as well as cardiac studies including chest radiograph and electrocardiogram with echocardiogram in many cases. When deficient enzyme activity is confirmed, molecular analysis is performed at one of several reference laboratories. When two or more mutations or variants are detected, it is recommended that parental testing be performed for phasing. However, the results of parental testing are not routinely collected by the screening program. Many clinicians obtain urine glucose or hexose tetrasaccharide (Glc4, Hex4) at the initial assessment and some include a full metabolic panel and brain natriuretic peptide (BNP). Over time, most centers have obtained fewer follow-up cardiac assessments than recommended on the algorithm on those infants with no initial evidence of cardiac disease. In particular, most infants who have the common c.-32-13T>G mutation do not undergo additional cardiac evaluation since the incidence of cardiac disease in association with this mutation has been shown to be very low [13]. Follow-up echocardiography for infants with other mutations is variable and at the discretion of the clinician.

Affected infants are classified as having infantile onset Pompe disease (IOPD) if they have evidence of cardiomyopathy in the first year of life. All other definitely affected infants are classified as having late onset Pompe disease (LOPD). The latter category includes infants found to have hypotonia, motor delay or muscle weakness in the first year of life. Infants who have no evidence of cardiac disease but have alpha-glucosidase activity in the affected range and have one pathogenic mutation and one variant of unknown significance (VUS) or two VUS are referred to as having possible LOPD or undetermined status. It is recognized that these infants may not be affected since the variants of unknown significance could be benign, but follow-up is recommended nonetheless, similar to what is provided to infants with definite LOPD. 

In the case of infants diagnosed with late onset Pompe disease (LOPD), or possible LOPD defined as low enzyme activity with one pathogenic mutation and one variant of undetermined significance (VUS) or two VUSs in trans, follow-up assessments are typically performed in the first year of life at 3 month intervals and include history and physical examination, developmental assessment, serum CK, and, in some cases, a complete metabolic panel and/or BNP, and urine Glc4. The undetermined cases are followed in the same manner as those with definite LOPD. For patients doing well, the interval between assessments is typically increased to 6 months to a year after one year of age. When LOPD or possible LOPD is diagnosed in an infant, older siblings born prior to the onset of newborn screening are routinely tested.

## 3. Results

A total of 684,290 infants were screened between 3 November 2014 and 30 September 2019. Three hundred and ninety-seven of these (0.06%), or 1 in 1724, screened positive for Pompe disease and were referred for diagnostic assessment. The outcome of the evaluation of these infants is seen in Table 1. A total of 29 infants had a definitive diagnosis of Pompe disease for a minimum incidence in the population of 1 in 23,596. Of these 29, 26 infants (90%) were found to have late onset Pompe disease. An additional 8 infants were defined as having an undetermined phenotype or “possible” Pompe disease. These were infants with low enzyme activity and two variants in the GAA gene, one or both of which were of unknown significance. Since it is likely that some of these infants will go on to develop Pompe disease, the incidence in the population is likely higher than the 1 in 23,596 calculated from the definite cases. Since screening was initiated, there have been no reports of infants diagnosed with Pompe disease who were missed by newborn screening. There have also been no infants with GAA activity in the affected range with only a single variant detected in the gene. 

Three infants had evidence of cardiomyopathy at the time of their initial assessment and were diagnosed with infantile onset Pompe disease (IOPD). All three were determined to be CRIM positive based on molecular analysis. Two were started on enzyme replacement therapy (ERT) with no immune modulation at 4 and 6 weeks of age. The third infant was also CRIM positive but had a mutation previously found to confer high risk for the development of high titer antibodies and had very severe cardiomyopathy requiring treatment in an intensive care unit from day 1 of life. He was treated with ERT beginning at 10 days of age and was simultaneously treated with rituximab, methotrexate, and intravenous immunoglobulin [14]. All three infants are currently doing well, all are ambulatory, and none of the three required any ventilatory support at the last follow-up. There were no infants who had a normal cardiac assessment initially who were later found to have evidence of cardiomyopathy.

Thus far, only one infant with LOPD has been started on ERT (Table 2, Case 25). This was an infant who, at the time of initial assessment, was found to have a CK of 555 international units per liter (IU/L) but no evidence of cardiomyopathy. At two months of age, head lag and mild hypotonia were noted but physical therapy assessment revealed a normal Alberta infant motor scale (AIMS) score at the 85th percentile for age. Multiple additional measurements of CK continued to be in the range of 550–650 U/L. Development appeared to progress normally for several months but by 9 months of age, significant hip laxity and gross motor delay were evident. The AIMS score had decreased to below the 5th percentile for age. A decision was made to initiate treatment and ERT was started at ten months of age, along with short term methotrexate [15]. After three months of therapy, the patient was making motor progress but continued to exhibit significant weakness so the dose of alglucosidase alfa was increased from the label dose of 20 mg/kg every two weeks to 40 mg/kg every two weeks. No other infants or children identified through newborn screening as having LOPD have been reported to have persistent clinical symptoms, although elevations of CK are common at diagnosis, as seen in Table 2.

For patients with more than two variants, clinical significance of all variants was not always evident. There were patients who had two pathogenic variants and either pseudodeficiency alleles or variants of unknown significance. All of the variants in the patients with definite or possible Pompe disease are listed in Table 2.

The results of the clinical and laboratory assessments performed on affected infants at the time of initial evaluation are seen in Table 2. It should be noted that these data are obtained from the consulting physicians by the Illinois department of public health and reporting may be incomplete in some cases. Due to privacy restrictions restricting release of identifying information, original records on all of these infants were not available to the authors. If a result is listed as NR (not reported), it may not have been done or may simply not have been reported, even if it was done. After the initial data were gathered following diagnosis, a follow-up questionnaire was sent annually to consulting physicians for each patient. The length of follow-up for the patients reported ranges from several months to 4 years.

## 4. Discussion

Pompe disease newborn screening has been successfully implemented in the state of Illinois over the past 5 years. As reported from other states [16], the overall incidence of Pompe disease was found to be higher than previously estimated, with the large majority of cases being of the late onset type. In addition, there were patients in whom a definitive diagnosis could not be established, who will require ongoing follow-up. 

The patients identified through newborn screening who are now on treatment have thus far had an excellent outcome and experience elsewhere has documented the benefits of treatment initiation prior to the onset of overt clinical symptoms [17]. Questions remain regarding the optimal follow-up of infants diagnosed with LOPD and the optimal time for initiation of treatment. It is anticipated that the collective experience of the many states now performing screening will shed light on these issues in the coming years. In the meantime, a method of systemic data collection to enable long-term assessment of outcomes and best practices is critically needed. Although there is an industry-sponsored registry for patients with confirmed Pompe disease [18], it is not ideal for the inclusion of asymptomatic patients, particularly those with genotypes that do not permit a definitive diagnosis (“possible” Pompe patients) and is not accessible by all treating physicians. A similar need exists for other disorders, including other lysosomal disorders, recently added to newborn screening since many are associated with phenotypes that may not become evident until much later in childhood or even in adult life.

## 5. Conclusions

Pompe newborn screening in the state of Illinois has been ongoing for 5 years and has led to the diagnosis of 3 infants with IOPD, 26 with LOPD, and eight with an undetermined diagnosis.

## Figures and Tables

**Table 1:** Outcome of follow-up in infants screen positive for Pompe disease (*n* = 395).

Category	Number of Infants Identified
Infantile Pompe disease	3
Late onset Pompe disease	26
Normal enzyme activity	234
Carrier ^1^	62
Pseudodeficiency ^2^	39
Phenotype undetermined ^3^	8
Loss to follow-up or refused	7
Died prior to follow-up ^4^	1
Pending	15

^1^ Infants with one pathogenic variant, or one VUS, with or without pseudodeficiency alleles were classified as carriers. ^2^ Infants in this category had only pseudodeficiency allelesc.1726G>A, c.2065G>A, and/or c.271G>A. 38 of 39 infants in this group were of Asian descent. ^3^ Infants in this category had one pathogenic variant and one or two VUS. ^4^ This was a premature infant who had multiple complications of prematurity but no findings to suggest Pompe disease.

**Table 2:** Follow-up data on infants with definite or “possible” Pompe disease.

Case	Genotype	Phenotype	GAA Activity ^a^ Result (nl)	CK (IU/L) Result (nl)	Urine Glc4 or Hex4 ^b^ Result (nl)	Cardiac Findings	Other Clinical Findings
1	c.2560C>T, c.1211A>T, c.2161G>C	IOPD	0.02 (>3.0)	1064 (35–232)	NR	HCM	Hypotonia
2	c.1437+1G>A, c.2227C>T	IOPD	0.8 (>3.88)	566 (32–250)	NR	HCM	Hypotonia; Motor delay
3	c.2560C>T, c.2459_2461del	IOPD	1.6 (>3.88)	3488 (30–279)	Glc4 14.9 (0.14–1.29)	HCM	Initial hypotonia ^c^
4–16	c.-32-13T>G homozygous	LOPD	0.0–2.8	153–669 (8/17 elevated)	See footnote ^d^	Normal ^e^	None
17	c.-32-13T>G, c.1655T>C	LOPD	0.8 (>3.0)	550	Normal	ASD	Mild hypotonia
18	c.-32-13T>G, c.2238G>C	LOPD	2.55 (>3.88)	86 (29–168)	Normal	None	None
19	c.-32-13T>G, c.1839G>A	LOPD	1.5 (>3.88)	641 (30–279)	Glc4 7.59 (0.14–1.29)	None	None
20	c.-32-13T>G, c.258DPC	LOPD	0.3 (>3.0)	NR	NR	None	None
21	c.-32-13T>G, c.2238G>C, c.2065G>A	LOPD	1.0 (>3.0)	NR	Hex4 41.6 (<20)	RVH on ECG; PFO on echo	None
22	c.-32-13T>G, c.2297A>G	LOPD ^f^	2.3 (>3.88)	168 (55–170)	Glc4 1.21 (0.08–1.37)	Normal	None
23	c.307T>G, c.1375G>C, c.271G>A	LOPD ^f^	1.6 (>3.88)	Normal	Glc4 2.0 (1.14–1.29)	Normal	None
24	c.1637-3_1637-4delinsG, c.1831G>A	LOPD	2.4 (>3.88)	93 (30–279)	Glc4 12.98 (0.14–1.29)	Normal	None
25	c.-32-12T>G, c.2219-2220delTG	LOPD ^g^	2.0 (>3.88)	555 (30–279)	Glc4 11.79 (0.14–1.29)	PFO	Hypotonia; gross motor delay
26	c.2238G>C, c.2242dupG	LOPD ^f^	2.9 (>3.88)	142 (30–279)	Glc4 6.54 (0.14–1.29)	Normal	None
27	c.2173delC, c.858+17-858+23delCGGGGCGG	LOPD	2.9 (>3.88)	272 (30–279)	NR	Normal	None
28	c.1121G>T, c.885C>T	LOPD	0.3 (>3.0)	NR	NR	Normal	None
29	c.307T>G, c.525delT	LOPD	0.5 (>3.0)	193 (55–170)	NR	Normal	None
30	c.655G>A, c.1418G>C	UND	3.0 (>7.4)	73 (39–308)	Hex4 11.2 (<20)	PFO	None
31	c.525delT, c.265C>T	UND ^f^	2.9 (>3.88)	74 (30–279)	Glc4 6.76 (0.14–1.29)	PFO ^h^	None
32	c.1942G>A, c.1346C>T, c.2065G>A, c.1726G>A	UND	0.2 (>3.0)	167 (39–308)	NR	Normal	None
33	c.664G>A, c.1346C>T	UND	0.0 (>3.88)	101 (30–279)	NR	Normal	None
34	c.726G>A, c.1357G>A	UND	0.7 (>3.0)	NR	NR	Normal	None
35	c.1631T>A, c.2509C>T, c.2065G>A	UND	UND	0.7 (>3.0)	NR	NR	Normal
36	c.307T>G, c.265C>T	UND	2.1 (>3.88)	152 (30–279)	NR	NR	Normal
37	c.1781G>A, c.1194+3G>C	UND	3.5 (>3.88)	571 (30–279)	Glc4 3.46 (0.14–1.29)	Normal	None

^a^ GAA activity units: pmol/punch/h. ^b^ Urine Glc4 or Hex4 activity units: mmol/mol creatinine. ^c^ Initial hypotonia and hypoventilation during sleep; resolved by 10 months of age. ^d^ 13 Cases: NR for 7; Glc4 5.42–9.83 (0.142–1.29) for 4; Hex4 6.8 and 13.2 (<20) for 2. ^e^ One case with left ventricular hypertrophy on ECG, with normal echo; all others normal. ^f^ Older sibling, born prior to newborn screening, has same genotype. ^g^ Symptomatic at 9 months of age, started on ERT. ^h^ Also had mildly dilated ascending aorta. ASD = Atrial septal defect. CK = creatine kinase. GAA activity = dried blood spot alpha-glucosidase activity determined at time of diagnostic evaluation. Glc4 = glucose tetrasaccharide. HCM = Hypertrophic cardiomyopathy. Hex4 = hexose tetrasaccharide. IOPD = Infantile onset Pompe disease. LOPD = Late onset Pompe disease. nl = Normal. NR = Not reported. PFO = Patent foramen ovale. RVH = Right ventricular hypertrophy. UND = Undetermined.
